# Digital light processing 3D printing of flexible devices: actuators, sensors and energy devices

**DOI:** 10.1038/s41378-025-00885-8

**Published:** 2025-03-19

**Authors:** Jiuhong Yi, Shuqi Yang, Liang Yue, Iek Man Lei

**Affiliations:** 1 Department of Electromechanical Engineering, University of Macau, Macao, 999078 China; 2 Centre for Artificial Intelligence and Robotics, University of Macau, Macao, 999078 China; 3 Smart Manufacturing Thrust, Hong Kong University of Science and Technology, Guangzhou, 511458 China

**Keywords:** Materials science, Engineering, Electrical and electronic engineering

## Abstract

Flexible devices are increasingly crucial in various aspects of our lives, including healthcare devices and human-machine interface systems, revolutionizing human life. As technology evolves rapidly, there is a high demand for innovative manufacturing methods that enable rapid prototyping of custom and multifunctional flexible devices with high quality. Recently, digital light processing (DLP) 3D printing has emerged as a promising manufacturing approach due to its capabilities of creating intricate customized structures, high fabrication speed, low-cost technology and widespread adoption. This review provides a state-of-the-art overview of the recent advances in the creation of flexible devices using DLP printing, with a focus on soft actuators, flexible sensors and flexible energy devices. We emphasize how DLP printing and the development of DLP printable materials enhance the structural design, sensitivity, mechanical performance, and overall functionality of these devices. Finally, we discuss the challenges and perspectives associated with DLP-printed flexible devices. We anticipate that the continued advancements in DLP printing will foster the development of smarter flexible devices, shortening the design-to-manufacturing cycles.

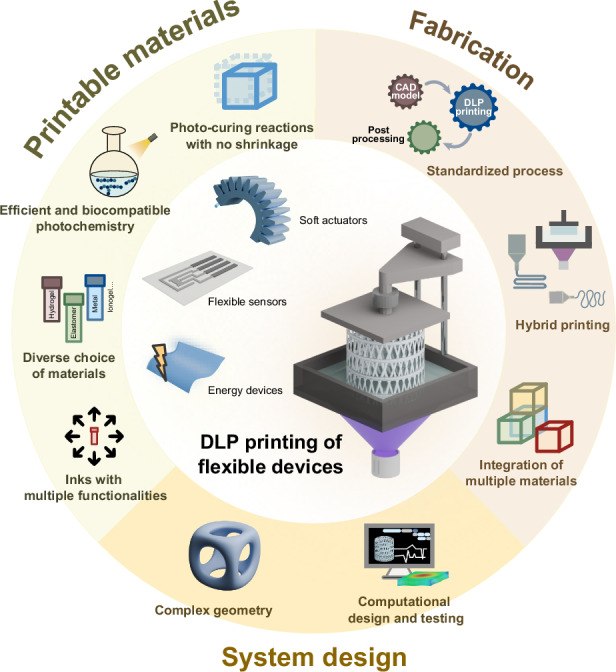

## Introduction

Flexible devices capable of forming conformal and safe human-machine interfaces with soft tissues have become increasingly pervasive in our lives, finding widespread applications in health monitoring, electronics and robotics^1–3^. These devices can undergo large mechanical deformations, such as bending, stretching and twisting, without compromising their stable performance. They offer the advantages of user comfort and lightweight designs, making them suitable for portable, wearable, and even implantable use^4^. However, traditional manufacturing approaches for flexible devices, such as casting, forging and lithography, often suffer from limited geometries, high costs, elaborate procedures, long fabrication times, waste production, and single functionality. Additionally, these techniques often lack compatible materials that combine easy processability, mechanical durability, biocompatibility and other advanced functionalities. As the demand for flexible devices continues to grow, a paradigm shift in the manufacturing approach is needed to enable simpler and more efficient fabrication processes that can incorporate different functional materials to advance these devices.

**Fig. 1:**
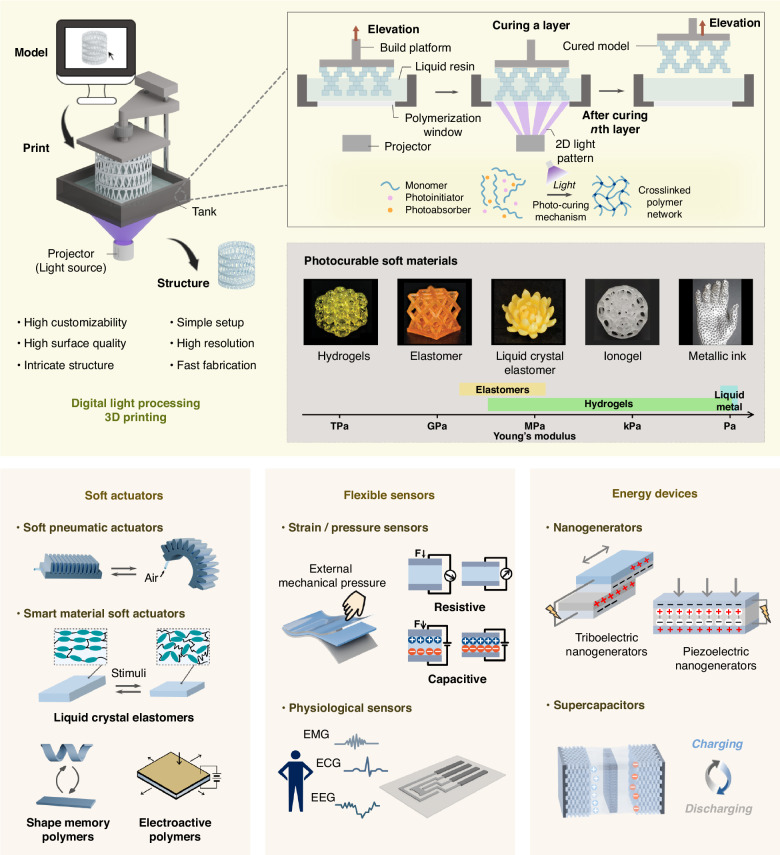
Overview of DLP 3D printing for flexible devices, including soft actuators, flexible sensors and energy devices. The figure illustrates the printing process of a bottom-up DLP 3D printing approach. EMG electromyogram, ECG electrocardiogram, EEG electroencephalogram. The photos of the photocurable soft materials are reproduced with permission from refs. ^62,70,98–100^

Recent progress in 3D printing has opened up exciting opportunities for flexible devices. 3D printing, also known as additive manufacturing, has emerged as a viable technology for material, engineering and life science research and industry, fundamentally reshaping how objects are created^5^. By enabling precise material patterning, 3D printing allows for customization, design flexibility and sustainability in manufacturing processes, expanding the possibilities of design, production and functionality. Various 3D printing techniques have been invented, each offering unique capabilities^6,7^. These include extrusion-based printing, such as fused deposition modelling (FDM) and direct ink writing (DIW), as well as vat polymerization 3D printing, such as stereolithography (SLA), digital light processing (DLP) and computer axial lithography (CAL). Among these techniques, DLP 3D printing, which involves projecting 2D patterns of light onto a vat of photopolymer resin layer-by-layer using a digital projector, offers indispensable potential in fabricating flexible devices (Fig. 1). Unlike FDM and DIW techniques, where resolution is primarily dependent on nozzle size, the resolution of DLP printing relies on the pixel size of the projector, allowing the production of highly intricate geometries with microscale features and smooth surface finishes. DLP also offers significantly faster printing speeds compared to other technologies, such as SLA, which employs a point-by-point curing mechanism. Additionally, DLP is compatible with a wide range of photocurable materials, including hydrogels, elastomers, ionogels, hard polymers, metals and ceramics, while FDM is limited to thermoplastics. These advantages are particularly beneficial for producing flexible devices that incorporate tailored and graded properties with multi-materials and multi-functions that were previously difficult to attain. For instance, the sophisticated and customized structures enabled by DLP improve the flexibility of devices, leading to higher sensitivity and better tissue adaptability. The ability to print conductive materials, such as ionoelastomers and ionogels, with DLP printing allows for the creation of highly conductive and functional components, enhancing the sensing precision of the devices. Furthermore, the simplicity and easy installation of DLP printers make this technology highly accessible for research laboratories and industries, positioning it as a versatile and facile tool for rapid prototyping innovative designs of flexible devices.

Despite previous reviews have focused on DLP 3D printing soft materials and vat polymerization^8–10^, there remains a lack of a timely review that comprehensively covers the wide spectrum of flexible devices enabled by DLP printing, particularly as the field rapidly evolves. In this review, we introduce DLP technology and discuss how DLP can be leveraged in the fabrication of functional flexible devices, emphasizing state-of-the-art advancements in soft actuators, flexible sensors, and flexible energy devices (Fig. 1). Finally, we discuss the challenges and future perspectives associated with this technology. We anticipate that this review will foster the future development of innovative flexible devices, broaden their application domains, and contribute to the advancement of 3D printing technology.

## Overview of DLP 3D printing

DLP 3D printing is an additive manufacturing technology that enables the rapid creation of high-resolution 3D macro- and micro-structures based on digital micromirror device (DMD) technology. A DLP 3D printer consists of several key components: a digital light projector as the light source, a vat filled with a liquid photocurable resin, a build platform where the resin is cured, and a mechanical motion system to control the movement of the build platform (Fig. 1). Entry-level DLP printers designed for laboratory or small-scale usage typically range from US$300 to $2000. A custom-developed DLP printer can be created with less than $1000^11^. The printer can be configured into top-down or bottom-up modes. In the bottom-up approach, the resin is cured from the bottom of the vat, offering the advantage of material saving. A classic DLP printing process begins with designing the desired 3D geometries using CAD software, such as AutoCAD and Onshape etc. These designs are then sliced into a series of 2D images using slicing software. During printing, the projector projects a 2D light image onto the vat of photocurable resin, enabling localized polymerization at high resolution. The build plate then moves upward to solidify the next layer. This process is repeated layer by layer until the full object is printed. Layer thickness, exposure time of each layer and light intensity are important parameters governing the fabrication efficiency and quality of the 3D printed parts, such as print resolution and surface roughness.

Compared to extrusion-based 3D printing, DLP printing benefits from high resolution, high throughput, and ease of manufacturing suspended features, reducing the consumption of support materials^12^. Its layer-wise printing mechanism allows for faster fabrication speeds than conventional extrusion 3D printing, and the print resolution can reach as low as 1 μm^13^. The physical and chemical properties of the resin highly influence the print quality. An ideal resin should have good stability, low viscosity, fast curing speed, minimal shrinkage during curing, and appropriate light transmission depth^14,15^. These resins can be prepared from photocurable materials, including hydrogels, elastomers, ionogels, hard polymers, metals and ceramics, with the addition of photo-initiators, photo-absorbers, which absorb excess light to improve resolution, and other additives for enhanced functionalities. Additionally, to ensure efficient printing and prevent the risk of print failures, a low viscosity of < 500 cps of the ink is required to efficiently fill the gap between the cured layer and the bottom of the vat^16^.

Recent technological advances have addressed traditional limitations of DLP printing, such as the brittleness of photocurable resins and challenges in creating heterogeneous constructs. For instance, grayscale digital light processing (g-DLP) has been developed, enabling facile preparation of structures with heterogeneous properties by spatially varying the brightness of the projected light^17,18^. Furthermore, recent explorations have realized multi-material 3D printing capability for DLP, enabling the fabrication of a single 3D object composed of multiple materials^19,20^. Continuous liquid interface production (CLIP) has been developed based on DLP^21^. This approach utilizes oxygen inhibition to prevent the liquid resin from curing and adhering to the bottom window during the upward movement of the print platform, enabling continuous fabrication without discrete steps and markedly accelerating the print speed. To promote the broader use of DLP printing, a portable DLP 3D printer has been developed based on a smartphone-powered projector and low-cost accessories^11^. These recent developments help achieve appealing properties and functions for flexible device and microsystem applications.

## DLP-printed soft actuators

Soft actuators, which utilize deformable materials, can interact with fragile objects and perform tasks with reduced risk of danger, while being highly resilient against mechanical damages^22^. Recent progress in material and architecture designs has innovated the development of soft actuators, and various types of soft actuators have been developed to meet a range of emerging applications, including wearable devices, haptic systems and micromanipulators^23^. These actuators are typically based on liquid crystal elastomers (LCEs), shape memory polymers (SMPs) or electroactive polymers (EAPs)^24^. With the capabilities of creating complex and high precise objects, DLP has presented a highly attractive approach for rapid prototyping soft actuators with advanced functionalities.

### Soft pneumatic actuators

**Fig. 2:**
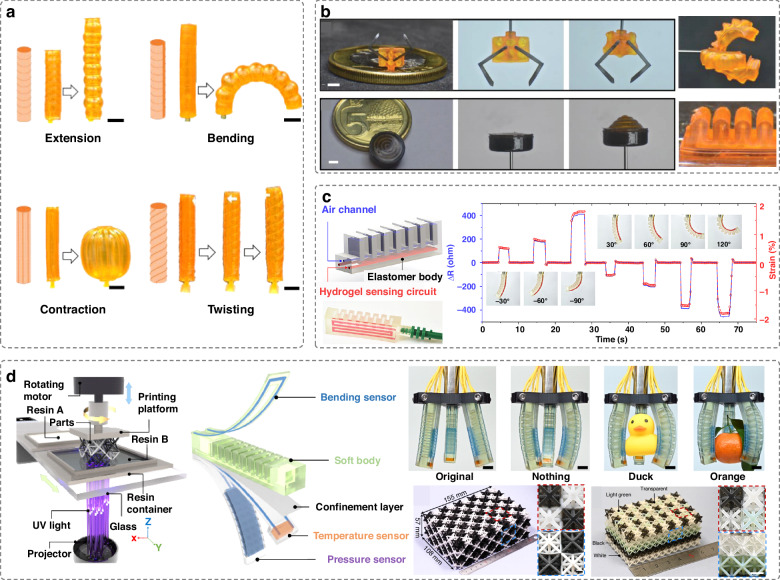
DLP printed soft pneumatic actuators. **a** g-DLP printed pneumatic actuators with heterogeneous stiffness, capable of performing extension, contraction, bending and twisting motions. Reproduced with permission from Springer Nature (2023)^26^. **b** A miniature soft gripper and a miniature soft inflatable actuator composed of stiff and soft materials. Reproduced with permission from Wiley (2019)^27^. **c** A DLP-printed soft pneumatic actuator with heterogeneous materials capable of detecting both positive and negative bending. Reproduced with permission from American Association for the Advancement of Science (2021)^28^. **d** Centrifugal multi-material 3D printing for producing soft actuators integrated with multiple sensors. Reproduced with permission from Springer Nature (2022)^20^

Soft pneumatic actuators powered by pressurized air in elastic bodies are an important category of soft actuation technologies. These actuators are lightweight, durable, and easy to install, providing stable performance^25^. DLP printing can fabricate microscale features in actuators that are difficult to achieve with traditional methods. Thus, fabricating actuators using DLP printing can offer new opportunities. By designing structures with variable properties and geometries, a range of actuation motions can be achieved.

g-DLP printing is a simple and effective approach capable of achieving varied material properties (e.g., color or stiffness variation) from a single resin material^17^. The technique harnesses grayscale light patterns to alter the intensity of the projected light intensities, thereby controlling the crosslinking density. Using this method, pneumatic actuators can be produced at high throughput with spatially customized stiffness ranging from highly stretchable organogels to stiff thermosets^26^. These actuators with varying stiffness can perform various motions, such as extension, torsion, contraction and bending (Fig. 2a). To facilitate the inverse design of the grayscale distribution, computational methods can be employed to allow for the creation of actuators tailored to specific mechanical properties that can achieve the intended actuation functions^18^.

Multi-material DLP 3D printing is another useful approach for achieving spatial control over material properties in heterogeneous 3D objects. This technique enables the development of hybrid pneumatic actuators with advanced functionalities. For example, composite actuators integrated with inextensible stiff materials and stretchable soft materials have been shown to perform various tasks, such as object picking using pneumatic soft grippers equipped with stiff claws (Fig. 2b)^27^. Additionally, hybrid pneumatic actuators composed of elastomers and conductive hydrogels can enable sensing capabilities, allowing for the detection of positive and negative bending (Fig. 2c)^28^. Traditional multi-material DLP printing has limitations of material contamination, slow print speed, small printing size and low function integration. Recently, a novel DLP centrifugal multi-material 3D printing approach has been proposed (Fig. 2d)^20^. This approach utilizes centrifugal force for non-contact cleaning of residual resin, preventing contamination while allowing for precise arrangement of voxels with varying composition, properties and functions. The method can accommodate a wide variety of materials, including hydrogels, hard polymers and ceramics, allowing the fabrication of soft multimodal actuators with five different materials. The actuators can integrate bending, pressure, and temperature sensing capabilities, capable of detecting objects with different shapes and temperatures.

To address the environmental problems caused by petroleum-based polymers in soft actuators, environmentally friendly alternatives have recently gained attention. By combining bio-based plant oil-derived elastomers with DLP printing, a fully printed, sustainable and pneumatically operated soft robotic gripper was created^29^. The bio-based elastomers exhibit adjustable mechanical properties by varying the biomass content and can be printed at high resolution, offering a promising replacement for petroleum chemicals in soft robotics. Porous structures in soft actuators can provide unique mechanical properties, such as high stretchability and compressibility. Bliah et al. proposed a 3D-printed soft pneumatic actuator with porous structures created using water-in-oil emulsion ink^30^. The porous structure enables high elongation-at-break value of up to 450%, and excellent reversible compressibility, even when compressed by 80%.

### Muscle-like actuators with LCEs

**Fig. 3:**
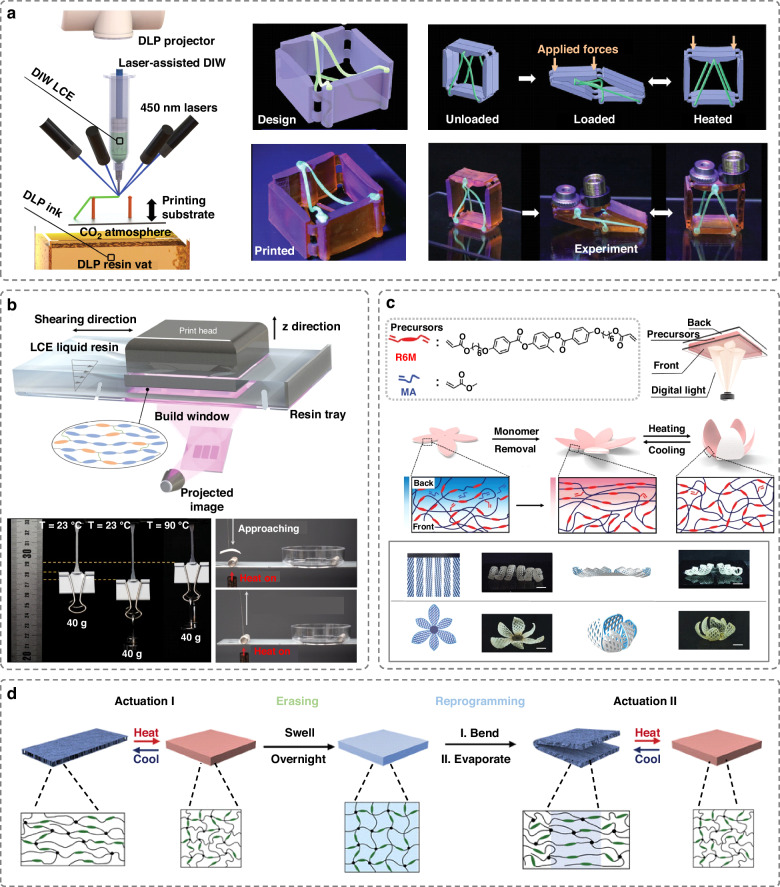
DLP-printed muscle-like actuators with LCEs. **a** A hybrid printed LCE-based actuator with tunable structural stability fabricated through DIW and DLP printing techniques. Reproduced with permission from Wiley (2022)^35^. **b** Shear alignment of LCEs caused by the shear induced by cyclic rotation of the resin tray. The printed actuators can achieve weightlifting and object grasping. Reproduced with permission from American Association for the Advancement of Science (2021)^36^. **c** Fabrication of LCEs actuators based on a through-plane light attenuation method, which induces crosslinking gradient for guiding a gradient of mesogen alignment during solvent evaporation. Reproduced with permission from Wiley (2021)^37^. **d** Actuators based on stretching-induced alignment of liquid crystal organogels to achieve erasable and reprogrammable actuation. Reproduced with permission from Wiley (2022)^38^

LCEs are attractive active materials for soft actuators. They can achieve rapid and programmable actuation through large shape transformation. LCEs are slightly crosslinked polymeric networks that can self-organize into liquid crystalline phases in response to external stimuli, such as thermal, optical, chemical and electric stimuli^31^. To enhance their actuation capability, aligning the initially random domain orientations of LCEs into a mono-domain is essential. DIW has been frequently explored for inducing alignment of LCEs. This approach can induce shear for alignment during extrusion, and a range of programmable actuating structures have been created^32,33^. However, owing to the line-by-line manner of material deposition in DIW, these structures are limited to simple actuation, such as simple planar shrinkage, bending and twisting. Recent research has demonstrated that combining DLP and DIW can enable the fabrication of soft actuators with new capabilities through a single printing job^34,35^. For example, functional LCE inks can be printed and aligned using DIW in conjunction with DLP printing to produce high-resolution structural support (Fig. 3a)^35^. The resulting hybrid active structures can achieve new actuation capabilities, such as freestanding LCE on-the-fly, tensegrity systems, weight-lifting actuators and actuators with tuneable stability.

Recent innovations have also made it possible to produce LCE soft actuators using DLP printing solely. By applying shear forces to align the LCEs during DLP printing and subsequently use UV light to lock the liquid crystal orientations, researchers have developed thermal-sensitive muscle-like actuators with a high orientational order of LCE (Fig. 3b)^36^. These actuators demonstrate versatile capabilities, such as object grasping, crawling locomotion, and weightlifting, and they exhibit thermally-induced optical transition, which can be utilized to develop self-sensing systems. However, creating non-continuous multilayered structures with oriented and non-oriented layers through mechanical shearing method can be time-consuming. To address this issue, a through-plane light attenuation technique has been proposed to rapidly fabricate LCE actuators with designable motions in 25 s (Fig. 3c)^37^. This method directs light onto a layer of precursor, leading to a crosslinking degree gradient that can guide the localized mesogen alignment during evaporation, as well as the overall shape of the actuators. Additionally, a study has reported that post-stretching treatment during solvent evaporation, can be applied to induce mesogenic alignment in DLP-printed liquid crystal organogels^38^. The alignments are erasable by reswelling the actuators, allowing for reprogrammable actuations (Fig. 3d).

### Other soft actuators based on smart active materials

**Fig. 4:**
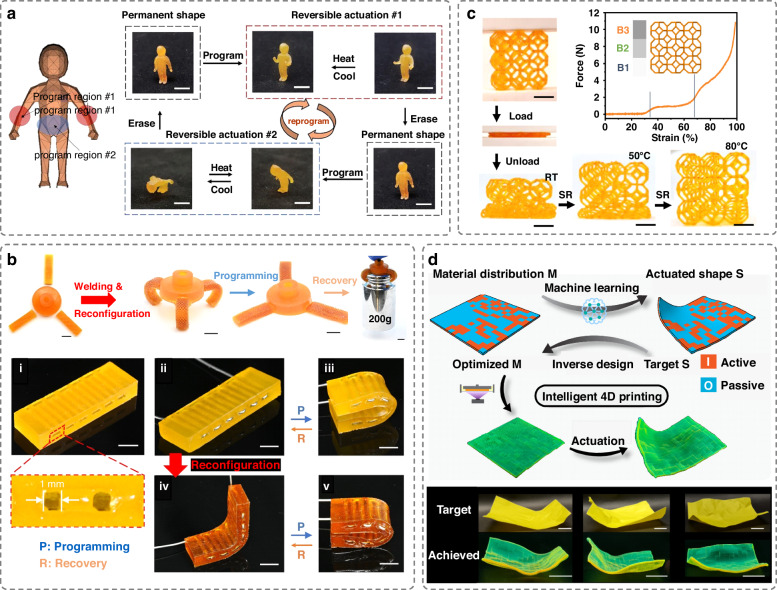
DLP-printed soft actuators based on SMPs. **a** A 3D printed actuator based on SMPs capable of exhibiting different reversible body movements. Reproduced with permission from Elsevier (2021)^44^. **b** 4D printed reconfigurable smart gripper and flat hinge based on mechanically robust CAN-SMPs. Reproduced with permission from American Association for the Advancement of Science (2024)^45^. **c** Cold-programmable 3D lattice structure produced using g-DLP. Reproduced with permission from Springer Nature (2023)^46^. **d** Machine learning model to facilitate inverse design of material distribution of 4D printed composites with active materials to attain the desired actuation shape. Reproduced with permission from Springer Nature (2024)^47^

In addition to LCEs, soft actuators can be produced with DLP using other stimuli-responsive soft materials, such as SMPs, EAPs, and magneto-responsive materials, which exhibit large shape transformation behavior under external stimuli. SMPs, which benefit from their simple activation mechanisms, high actuation forces and tuneable moduli varying across three orders of magnitude, have been extensively used in developing stiffness-variable robots^39,40^. Various DLP-printed SMP-based responsive structures have been developed, possessing features such as fatigue resistance^12^, rapid fabrication capabilities with material heterogeneity^41^, and NIR light-sensitive shape memory structures for myocardial regeneration, etc^42^. Conventional SMPs are limited to one-way transformations, allowing for the recovery of their permanent shape, but not their temporary shape. To address this limitation, recently emerged SMPs have incorporated advanced material designs, such as integrating two crystalline phases (Fig. 4a) or LCEs in SMPs, to enable reversible actuation between different shapes^43,44^. By leveraging the advantages of both LCEs and SMPs, LCE-SMP composite actuators can achieve rapid and reversible shape changes, while maintaining high stiffness in the actuated state^43^. To enhance configurability, covalent adaptable networks (CANs) can be integrated into SMPs to create weldable and highly complex 3D shape-memory structures, such as shape-changeable grippers and smart hinges, through DLP printing (Fig. 4b)^45^. The structures can be reversibly reconfigured to multiple permanent shapes, capable of fulfilling a variety of tasks with one printed structure. Conventional DLP-printed SMP structures typically exhibit shape changes through a heating process. Recently, DLP-printed actuators with cold-programming capabilities have been developed, capable of programming the shapes at room temperature (Fig. 4c)^46^. Utilizing the stress-induced relaxation effect in polymers, these structures can be deformed into a temporary shape without the need for increasing temperature, providing a simple yet convenient method for programmable shape morphing. To achieve desired actuation shapes in 4D-printed active composites, a study has exploited a machine learning algorithm to facilitate inverse designs^47^. The algorithm can determine the material distribution of active materials, such as SMPs and passive materials within a construct to achieve intended actuation shapes (Fig. 4d).

EAPs are candidates for electro-responsive soft actuators as they can change size or shape when subjected to electric field stimuli. EAPs can be classified into ionic EAPs and electronic EAPs. Taking advantage of the low voltage actuation capability of ionic EAPs (< 5 V), hydrogel actuators, such as valve actuators, have been made from ionic EAPs using DLP, demonstrating large deformation, high actuation speed and stable actuation performance^48^. In contrast, electronic EAP actuators, such as dielectric elastomer actuators, can generate larger actuation forces than ionic EAPs, but they typically require high electrical voltages (> 1 kV)^49^. To improve output performance, DLP has enabled the creation of multilayer dielectric elastomer actuators, significantly enhancing output performance compared to single-layer designs. These actuators can serve as vibrotactile devices for prosthetic applications^50^. Additionally, magnetic actuators have been fabricated with DLP printing using magneto-responsive materials. These actuators can change shapes in response to external magnetic fields, enabling motions, such as rolling, stretching, folding/unfolding and grasping^51,52^.

## DLP-printed flexible sensors

### Flexible strain/pressure sensors

Flexible strain or pressure sensors, which detect mechanical deformations through electrical signals, have found widespread applications in electronic skins, human motion monitoring, human-machine interaction, and healthcare inspection^53^. Broadly, these sensors can be classified into four categories based on their working mechanism: resistive, capacitive, piezoelectric, and triboelectric sensors. DLP printing has emerged as an efficient approach for fabricating resistive and capacitive sensors, as this technique can enhance performance and simplify the preparation process by enabling precise and customizable structuring of functional materials^54^. So far, a variety of materials have been developed for DLP printing of these sensors, including ionic hydrogels^55–59^, nanoscale materials^60^, cryogels^61^, elastomers^62–65^, ionogels^66^, and liquid metal^67^. Moreover, various structures, such as gyroid, octahedral, honeycomb, star, pyramid and trigonal beam-shaped designs^58,59^, have been successfully fabricated using DLP, without the requirement of support materials. These designs significantly improve deformability, leading to sensors with higher sensitivity and a wider sensing range.

#### Flexible resistive sensors

**Fig. 5:**
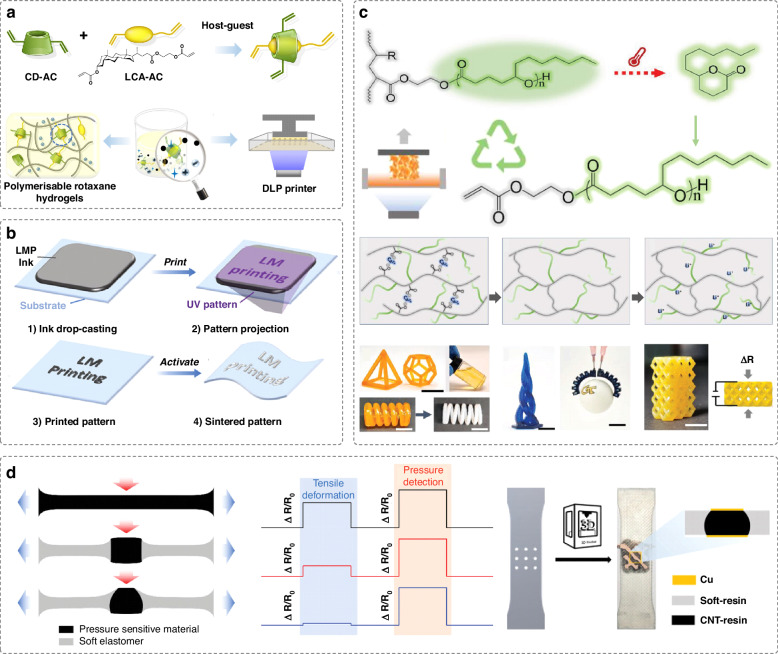
DLP-printed flexible resistive strain/pressure sensors. **a** A DLP printable polymerizable rotaxane hydrogel assembled by precise host-guest recognition that can be used for creating resistive sensors with fatigue resistance. Reproduced with permission from Springer Nature (2023)^55^. **b** DLP printing of conductive liquid metal patterns, which can be used for various applications, such as resistive sensors, heaters and electrodes for electrography. Reproduced with permission from Wiley (2024)^67^. **c** A resistive sensor and functional construct produced based on recyclable ion-conductive Δ-Valerolactone thermoset ink. Reproduced with permission from Wiley (2024)^71^. **d** Structural design of a flexible pressure sensor to realize tensile strain insensitivity. Reproduced with permission from Wiley (2024)^73^

Owing to their simple read-out mechanism, ease of fabrication, high linearity, and low power consumption, resistive sensors are popularly used in flexible electronics^68^. These sensors function by measuring changes in electrical resistance caused by mechanical forces, such as stretching, bending, and rolling. Designing DLP-printable material formulations can significantly improve the functionality of resistive sensors. Hydrogels are one of the promising candidates because of their good biocompatibility, ionic conductivity and tissue-like wetness. However, like many conventional DLP-printed materials, acrylate-based photocurable materials tend to be brittle. In additional, conventional hydrogels are mechanically fragile. These have significantly limited their long-term and practical applications^69^. Recent studies have introduced innovative material designs aimed at enhancing their mechanical properties. For instance, incorporating nanoclay into photocurable ionic conductive hydrogels has resulted in DLP-printed resistive sensors with reinforced stretchability and tensile strength^57^. By designing ionic conductive hydrogels with self-healing capabilities, resistive sensors can recover their strain sensitivity after damage^58^. These sensors can achieve high-pressure sensitivity with a detection limit below 1 Pa, facilitated by the complex-shaped designs enabled by DLP. Recently, DLP-printed resistive strain sensors have been developed using a novel polyrotaxane hydrogel formed by host-guest complexes of acrylated β-cyclodextrin and bile acid derivates (Fig. 5a)^55^. This system exhibits topological networks with mobile junctions after photocuring, endowing the sensors with excellent stretchability, self-healing, superior fatigue resistance and repeatable adhesion. These sensors can be printed into mesh-like structures, enhancing their conformal interface with human skin for monitoring movements, such as finger pressing and joint bending. Apart from resistive sensors, these hydrogels can be utilized to print electrodes for real-time tracking of human electrocardiogram signals.

Hydrogels are prone to solvent evaporation, causing unstable performance in hydrogel devices. Recent advances in ionoelastomers^62,63^, ionogels^66,70^ and conductive elastomers, such as multiwalled carbon nanotubes/elastomer composites^64,65^, present new opportunities for DLP-printed flexible sensors. These materials help mitigate evaporation issues, thereby enhancing the electrical stability of the devices. Liquid metals also emerge as attractive candidates for flexible sensors as they can improve electrical conductivity, while providing excellent stretchability. Wu et al. pioneered the DLP printing of liquid metal particle inks to produce highly conductive crosslinked liquid metal patterns for the first time (Fig. 5b)^67^. The ink is formulated with polymer precursors and functionalized particles of eutectic gallium indium (i.e., a low-toxicity liquid metal) with oxide skins to reduce surface tension. This innovative ink allows for the facile production of various electrode patterns in less than 10 s of UV projection. The conductive patterns can be utilized in diverse applications, including not only epidermal strain sensors, but also electrography, finger contact sensors, stretchable displays, resistive heaters and humidity sensors.

Recyclable devices made from bio-based materials have garnered increasing attention due to growing global sustainability concerns. Recent literature has developed a bio-based, recyclable and ion-conductive Δ-Valerolactone thermoset ink that can be applied to sustainable DLP-printed strain sensors (Fig. 5c)^71^. These sensors can be depolymerized and repolymerized, demonstrating the potential for closed-loop circular DLP printing. With DLP printing, sensors can be architected with unique geometries to enable novel functionalities. For example, inspired by the pressure-withstanding ability of deep-sea organisms, DLP has been employed to fabricate resistive sensors featuring hollow interlocking structures. These sensors are capable of exhibiting a high sensitivity of 1.74 kPa, even under 30 MPa of static hydrostatic pressure, showing potential for deep-sea applications^72^. Sensors that can accurately distinguish between different types of deformations, such as compression, bending and stretching, are important. A recent study developed a resistive pressure sensor composed of soft and hard segments using DLP printing (Fig. 5d)^73^. With an optimized geometrical design, this sensor can selectively detect compression force, exhibiting high sensitivity to compression force while remaining insensitive to tensile strain.

#### Flexible capacitive sensors

**Fig. 6:**
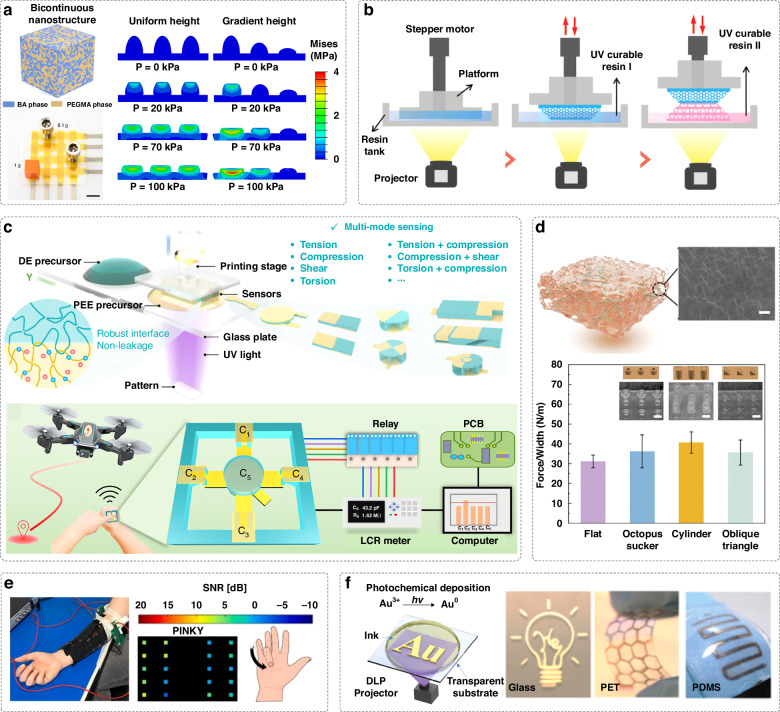
DLP-printed capacitive strain/pressure sensors and electrodes for electrography. **a** Capacitive sensors featuring DLP-printed domes with a gradient height to enable a high sensitivity, produced from a highly conductive and stretchable nanostructured ionogel. Reproduced with permission from Springer Nature (2024)^66^. **b** Capacitive sensors with stable bonding interface produced from acrylic-based dielectric materials and conductive hydrogels using a dual-material DLP printing strategy. Reproduced with permission from Wiley (2019)^76^. **c** An ionotronic capacitive sensor with multi-mode sensing capabilities that can be harnessed to control the flight of a drone wirelessly. Reproduced with permission from Springer Nature (2023)^78^. **d** Capacitive sensors composed of silk fibroin-based biocompatible hydrogel electrodes with different surface structures for superior adhesive properties. Reproduced with permission from Wiley (2024)^56^. **e** Multiple electrodes made from PSS:PEDOT eutectogels can be sewn into the textile for body surface potential mapping of the forearm. Reproduced with permission from Elsevier (2024)^82^. **f** DLP printed Au electrodes based on anion-assisted photochemical deposition with high conductivity and conformability for electrophysiological signal monitoring. Reproduced with permission from Wiley (2022)^83^

Capacitive sensors typically consist of a dielectric material sandwiched between two electrodes. These sensors work by detecting changes in electrical capacitance caused by the reduced distance between electrodes during deformation^74^. To improve the sensitivity of capacitive sensors, porous or foam-like microstructural designs are often employed. These structures can increase the dielectric constant and reduce the elastic modulus of the devices, thus improving deformability and sensitivity. DLP is a capable technology for producing precise and fine porous structures, thereby improving sensitivity. For example, capacitive sensors, composed of DLP-printed stretchable hydrogel electrodes with microstructural designs, can achieve high sensitivity (up to 0.91 kPa^−1^) and a low strain detection limit, capable of detecting both static and dynamic pressures^75^. Ionoelastomer-derived capacitive sensors with a gap architecture and a DLP-printed microcircuit design demonstrate sensitivities over 44 times higher than their bulky counterparts^62^. To address the low ionic conductivity limitation of traditional ionoelastomers (10^−5^ ~ 10^−2 ^S/m), a capacitive sensor composed of conductive DLP-printed nanostructured ionogels has been proposed, enabling high ionic conductivity (> 3 S/m), stretchability (> 1500%), low hysteresis and high-resolution printability (Fig. 6a)^66^. By architecting the ionogel in dome shapes with gradient heights, these capacitive sensors have superior sensing performance. They exhibit an outstanding sensitivity of 15.1 kPa^−1^, allowing for real-time monitoring of physiological signals, including deep breathing, swallowing and pulsation across a wide temperature range.

Dual-material printing strategies are efficient one-step methods for fabricating capacitive sensors by alternately printing dielectric components and photocurable conductive hydrogels to create sandwich structures. The process does not require adhesion or assembly steps, realizing rapid fabrication. For instance, utilizing acrylic-based resins for both dielectric and conductive hydrogel components, capacitive sensors with stable interfaces between components can be achieved^76,77^. Similarly, the hydrogel electrodes can be printed with microstructures to enhance sensitivity. Yin et al. show that using a dual-material printing method, the resulting capacitive sensor demonstrates good repetition durability, excellent sensitivity, fast response speed and low hysteresis that can detect human physiological signals and human-machine interactions in real-time (Fig. 6b)^76^. Moreover, to enable the simultaneous differentiation of multiple types of mechanical stimuli, ionotronic sensors have been produced using DLP multi-material 3D printing with a polyelectrolyte elastomer and a dielectric elastomer. By carefully designing the materials and structures of the sensors, these sensors can simultaneously perceive different mechanical stimuli without mutual signal interferences, including tension, compression, shear, torsion, combined tension and compression, combined compression and shear, and combined torsion and compression (Fig. 6c)^78^. These sensors can be integrated to create a wearable device for remotely controlling a drone.

Conformity of flexible devices to skin is crucial in wearable applications, as it directly affects the stability and quality of signal measurements. DLP printing facilitates facile fabrication of user-specific wearable sensors. The geometries of these sensors can be easily tailored to conform to the detection locations, such as fingers and wrists, enabling real-time signal reflection of body movements^59^. Additionally, capacitive sensors can be designed with biocompatible hydrogel electrodes possessing sweat-releasing channels produced using DLP printing^56^. These sensors directly adhered onto skins, realizing comfortable adhesion and reliable monitoring of body motions with high repeatability and stability (Fig. 6d).

### Flexible electrodes for electrophysiology measurements

Flexible electrodes are crucial components in flexible electronics. They are capable of acquiring various electrophysiological signals for continuous health monitoring and detection of abnormal health issues. However, current flexible electrodes often suffer from poor contact with skin and low signal-to-noise ratios^79^. DLP printing has been leveraged to improve the skin conformability of flexible electrodes. By engineering electrodes with innovative surface structures, such as octopus-inspired suction cup structures^80^, mushroom-like protuberance structures^81^, and wavy surface patterns^82^, greater adhesion and conformability can be achieved. These designs help eliminate air gaps, ensuring the reliability of the recorded electrophysiological signals. Serrano et al. demonstrate that electrodes with wavy surface patterns made from PSS:PEDOT eutectogels can achieve full skin conformability for forearm electromyogram measurements^82^. By embedding multiple electrodes into a textile, high-resolution body surface potential mapping of the forearms can be generated, accurately detecting individual finger movements, which can be used to translate sign language (Fig. 6e).

Gold is a noble metal known for its excellent chemical stability, conductivity and biocompatibility, making it ideal for flexible electronics applications, particularly in implantable devices. A recent study has enabled the DLP printing of gold electrodes, providing a one-step, annealing-free method for patterning gold structures with high resolution^83^. By utilizing an anion-assisted photochemical deposition approach, gold precursor inks containing Au^3+^ ions can be facilely converted and reduced into a gold nanoparticle network of Au^0^ with customized shapes through UV light projection (Fig. 6f). Owing to their high conductivity (10^7 ^S/m) and conformability, the electrodes are highly suitable for signal acquisition of electrocardiograms, electromyograms, and electroencephalograms.

## DLP-printed flexible energy devices

As wearable and flexible electronics become more prevalent, there is a growing demand for new energy devices that are lightweight, miniaturized, flexible, durable, low-cost, and eco-friendly. Power sources, such as nanogenerators and supercapacitors (SCs), can now be designed to be flexible or even stretchable. These devices can seamlessly integrate with diverse geometries, such as the surfaces of organs or tissues, enabling untethered operation. However, producing these devices using traditional methods, such as spin coating and chemical vapor deposition, often faces challenges related to process complexity, extended production times, high costs and large amounts of material waste. Additionally, these manufacturing processes often restrict the miniaturization and customization of devices. DLP printing thus presents new opportunities for enabling new functionalities of flexible energy devices.

### Flexible nanogenerators

Nanogenerators convert various forms of low-intensity discontinuous mechanical stimuli from the environment into electrical power. They represent promising candidates for generating sustainable and renewable energy for wearable electronics. Two common approaches for energy harvesting are triboelectric nanogenerators (TENGs) and piezoelectric nanogenerators (PENGs).

**Fig. 7:**
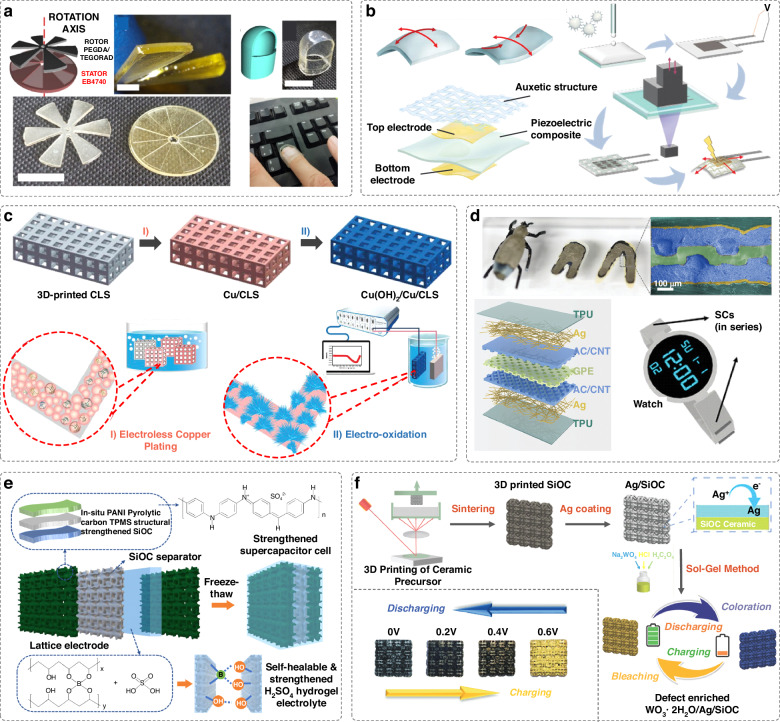
DLP-printed energy devices. **a** A 3D-printed rotating TENG and a TENG with thimble geometry for mechanical energy harvesting. Reproduced with permission from American Chemical Society (2023)^87^. **b** An auxetic structure-assisted PENG that can harvest energy in the bending mode. Reproduced with permission from Wiley (2023)^88^. **c** A SC with adjustable capacitance based on urchin-like Cu(OH)_2_ lattice electrodes fabricated by DLP and electro-oxidation method. Reproduced with permission from Wiley (2019)^91^. **d** A structural SC in the shape of a watchband produced through DLP and dipping processes. The device is designed for lightweight and miniaturization, suitable for powering an electronic watch. Reproduced with permission from Wiley (2023)^93^. **e** An impact-resistant SC with self-healable hydrogel electrolyte-infused lattice electrodes that can work under harsh environments. Reproduced with permission from Wiley (2024)^95^. **f** A 3D structured electrochromic SC can visually monitor the energy storage levels by a color change. Reproduced with permission from Wiley (2021)^96^

TENGs generate electricity based on the triboelectric effect (i.e., contact-induced electrification effect), which induces and transfers electrostatic charges when two different materials come into contact or slide against each other^84^. The structural designs, surface texture and material selections of these devices significantly influence their charge generation capacity^85^. Therefore, DLP printing, which can alter the device structures, can be harnessed to enhance the performance of nanogenerators. DLP printing has been used to create TENG devices with a biomimetic-villus structure, which increases surface area^86^. By incorporating polytetrafluoroethylene powder as triboelectric materials, this design can result in a fivefold increase in electric power output performance. Moreover, this structure can function as an eco-friendly durable dust-absorption system, generating electrostatic charges during dust collection. To demonstrate the potential of DLP for fabricating TENGs, a recent study has comprehensively evaluated the performance of common DLP-printable materials as triboelectric layers and defined a triboelectric series for these materials^87^. Using these materials, devices with increased geometrical complexity and enhanced energy conversion performance can be entirely produced with DLP. Examples include a rotating TENG device and a printed TENG thimble, which can be inserted into a finger and generate electrical voltage when pressed on different materials, such as a keyboard (Fig. 7a).

Different from TENGs, PENGs generate electricity through mechanical deformation by internal polarization in piezoelectric materials. Polymer-based PENGs are advantageous for their flexibility and lightweight properties. However, most polymer-based PENGs, such as polyvinylidene fluoride (PVDF)-family polymers, are limited to energy harvesting through compression using the d33 operation mode, where deformation occurs in the same direction as the polarization of the piezoelectric materials. The non-stretchable nature of PVDF-family polymers makes it difficult to develop PENGs that can harvest energy via stretching or bending modes. In this regard, DLP printing can enable innovative structural designs to address the limitations of these non-stretchable materials. For example, auxetic structures can be fabricated with DLP and integrated into PENGs, enabling the harvesting of bending energy by transforming bending deformation into stretching deformation (Fig. 7b)^88^. By measuring the output voltage, the device can precisely sense bending angles and monitor human motions.

### Flexible supercapacitors

As an indispensable class of energy storage devices, SCs possess the advantages of high-power density, fast charge/discharge rate, excellent cycling stability, low cost and reduced pollution to the environment^89^. However, compared to commercial Li-ion batteries, their low energy density is still a major drawback. With the increasing demand for sustainable and renewable energy systems for wearable electronics, Internet-of-Things, etc., developing novel SCs with high capacitance, good mechanical performance, miniaturization, and customization is pressingly required.

SCs typically consist of current collectors, electrodes, an electrolyte and a separator. Constructing 3D structured electrodes with periodic pores is an efficient strategy to improve the electrochemical performance of SCs significantly. These structures increase the electrode-electrolyte interface, therefore facilitating the number of ions adsorbed on the electrode surface^90^. Various studies have documented the use of DLP to develop advanced electrode structures for SCs. For instance, Chang et al. reported a SC with adjustable capacitance based on urchin-like Cu(OH)_2_ lattice electrodes, fabricated using DLP and an electro-oxidation method (Fig. 7c)^91^. The high surface area of this electrode lattice considerably improved both electrochemical performance and mechanical properties, with capacitance and mechanical strength increasing as the coordination number of the lattice increased. Similarly, DLP has enabled the fabrication of porous graphene-based hierarchical composite electrode lattices for quasi-solid SC devices^92^. These hierarchical porous micro-lattice structures, ranging from nm to mm, allow the SCs to achieve high energy density (0.008 mWh/cm^2^), high areal capacitance (57.75 mF/cm^2^), rate capability and long lifespan.

The ability to customize the internal structure of SCs is crucial for optimizing device performance, as it allows for the design of shortened diffusion pathways for electrons and ions. A new method, which enables the customization of both the internal and external architectures of SC devices, can be achieved through DLP printing of a 3D electrolyte template, followed by dip-coating of the electrode, current collector and packaging materials^93^. The customized internal structure significantly enhances the areal capacity than that of planar stacked configurations. Additionally, the customizable external shapes facilitate easy integration with other electronics. For instance, they can be printed in the shape of a watchband to power electronic watches, offering the potential for weight reduction and miniaturization (Fig. 7d).

Moreover, through the synergy of DLP printing and innovative printable materials, SCs can be developed with advanced properties while meeting electrochemical demands. Stretchable micro-SCs with DLP-printed octet-truss ionic hydrogel electrodes show enhanced interfacial contact and capacitance, even at low temperatures (i.e., −30 °C), and can be easily integrated with flexible electronics^90^. DLP-printed gyroidal 3D carbon foams, exhibiting hierarchical porosity that promotes electron/ion transportation and capacitance, can provide high compressive strength^94^. To improve the safety of SCs in extreme environments, Zhou et al. fabricated an impact-resistant, load-bearable, self-healable and ready-to-use SCs based on a hydrogel-infused lattice (Fig. 7e)^95^. The DLP-printed porous current collectors provide mechanical protection. Under harsh conditions, the SC can maintain operational integrity and self-healing property after electrolyte damage. Additionally, a study has demonstrated the development of smart SCs capable of visually monitoring the energy storage levels through reversible color changes (Fig. 7f)^96^. These devices were fabricated with DLP printing of ceramic micro-scaffolds integrated with electrochemical and electrochromic tungsten oxide hydrates.

## Conclusion and outlook

Over the last decade, DLP 3D printing has been increasingly embraced by the research community as an effective and accessible additive manufacturing approach for fabricating microsystems and devices. In this review, we discuss the latest advancements in DLP printing for flexible devices, including soft actuators, sensors, and energy devices. We emphasize the capabilities of architectural designs, material innovations and technological advances to enable the creation of devices with unique properties and enhanced performance. For example, recent novel developments in DLP printable photocurable materials, including hydrogels, elastomers, ionogels, and liquid metals, have led to flexible devices with distinct functionalities, such as extraordinary sensing sensitivity. Additionally, customized devices with sophisticated architectures can be easily created through DLP without the need for costly setups and labor-intensive processes. Emerging techniques, including g-DLP, multi-material DLP 3D printing, and DLP-based 4D printing, have successfully facilitated precise control over material property distribution within a single structure or adaptability over time, further enhancing the multifunctionalities and sensitivity of flexible devices.

**Fig. 8:**
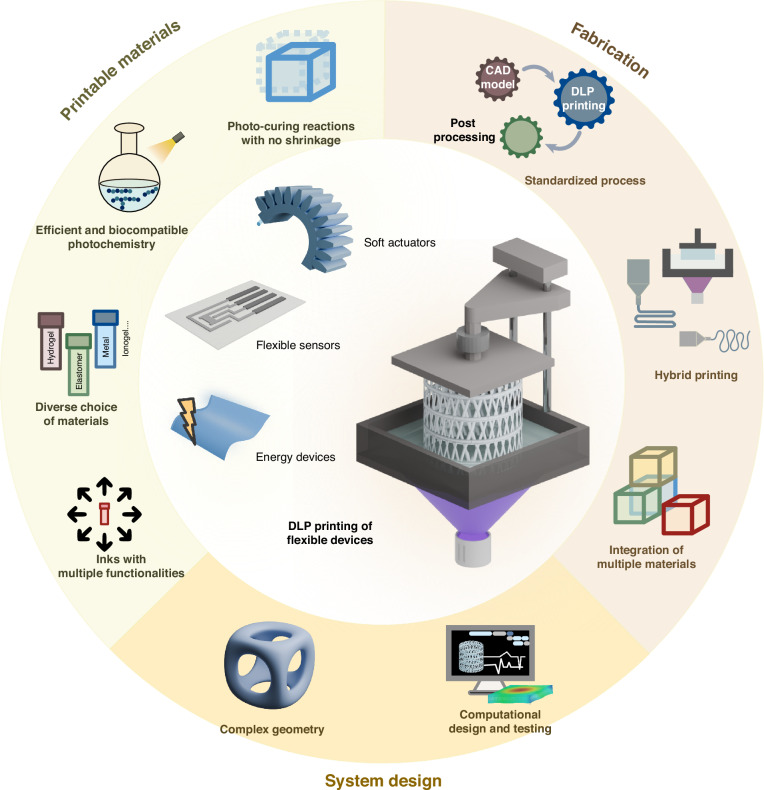
Perspective on DLP printing of flexible devices

Despite the above advancements, DLP printing has yet to achieve widespread acceptance for developing flexible devices, particularly at industrial scales, due to several key challenges. Figure 8 denotes the perspective on DLP printing of flexible devices. One of the most important obstacles is associated with the non-standardized manufacturing approach, which hinders the reproducibility and consistent performance of the devices. Additionally, the functionality of DLP-printed systems is limited by a restricted choice of printable materials. Compared to other 3D printing technologies, such as FDM and DIW, the spectrum of commercially available photocurable materials for DLP printing is generally more restricted, with many lacking biocompatibility. Breakthrough in photochemistry will broaden the choice of materials for DLP printing and, thus, the functionalities of the devices. For example, exploring biofriendly photo-polymerization chemistry, such as visible light photocrosslinking with Ruthenium^97^, is essential for developing future biocompatible devices or flexible implants with cells using photo-based 3D printing. Efforts should be devoted to developing innovative photocurable inks that are capable of integrating multiple properties, such as mechanical stretchability, softness, durability, conductivity and recyclability, as achieving these simultaneously is often challenging. Successfully incorporating these properties will broaden their applications. The development of highly reactive photoinitiators and UV curing systems, which facilitate rapid and controlled polymerization, can reduce printing time and energy consumption. Additionally, innovative designs of photochemistry can enable on-demand light-induced degradability of devices, facilitating their environmentally friendliness after use. Addressing the shrinkage issue during monomer curing is essentially for improving printing precision. Developing photo-absorbers compatible with various inks and printing light wavelengths can lead to the efficient creation of constructs with high resolution. Furthermore, hybrid 3D printing approaches, combining DLP printing with other fabrication techniques (e.g., other 3D printing methods or electrospinning), should be further explored. This combination would leverage the strengths of each method, empowering the all-in-one production of flexible electronics with a diverse range of materials, reduced production time, lower cost and scalability, while preserving the high-resolution advantage of DLP printing. Lastly, the current designs of DLP-printed devices are relatively simplistic. Complex materials geometries should be designed using computational modelling to optimize performance metrices, such as sensing sensitivity, energy conversion and actuation capacities. Improvements in instrumental designs should enable more intricate designs through DLP 3D printing.

Looking forward, addressing these limitations will present numerous opportunities and design freedom for flexible devices. We anticipate that the continued growth of DLP 3D printing technology, along with advancements in soft materials and a strong demand for flexible devices, will propel the development and usage of smart, high-performance flexible devices.
